# Progressive Resistance Exercise and Parkinson's Disease: A Review of Potential Mechanisms

**DOI:** 10.1155/2012/124527

**Published:** 2011-11-24

**Authors:** Fabian J. David, Miriam R. Rafferty, Julie A. Robichaud, Janey Prodoehl, Wendy M. Kohrt, David E. Vaillancourt, Daniel M. Corcos

**Affiliations:** ^1^Department of Kinesiology and Nutrition, University of Illinois at Chicago, Chicago, IL 60612, USA; ^2^Graduate Program in Neuroscience, University of Illinois at Chicago, Chicago, IL 60612, USA; ^3^Division of Geriatric Medicine, University of Colorado School of Medicine, Aurora, CO 80045, USA; ^4^Department of Applied Physiology and Kinesiology, University of Florida, Gainesville, FL 32611, USA; ^5^Department of Neurology, University of Florida, Gainesville, FL 32610, USA; ^6^Department of Bioengineering, University of Illinois at Chicago, Chicago, IL 60612, USA; ^7^Department of Physical Therapy, University of Illinois at Chicago, Chicago, IL 60612, USA; ^8^Department of Neurological Sciences, Rush University, Chicago, IL 60612, USA

## Abstract

This paper reviews the therapeutically beneficial effects of progressive resistance exercise (PRE) on Parkinson's disease (PD). First, this paper discusses the rationale for PRE in PD. Within the first section, the review discusses the central mechanisms that underlie bradykinesia and muscle weakness, highlights findings related to the central changes that accompany PRE in healthy individuals, and extends these findings to individuals with PD. It then illustrates the hypothesized positive effects of PRE on nigro-striatal-thalamo-cortical activation and connectivity. Second, it reviews recent findings of the use of PRE in individuals with PD. Finally, knowledge gaps of using PRE on individuals with PD are discussed along with suggestions for future research.

## 1. Introduction

The standard treatment for Parkinson's disease (PD) is pharmacologic treatment with levodopa, a precursor to dopamine. However, continued treatment with levodopa is associated with motor side effects such as dyskinesias and motor fluctuations. Until an oral formulation of levodopa without the accompanying motor side effects is formulated, surgical options offer some relief. Typically, surgery is reserved for when the disease and the side effects due to medication are severely disabling. Currently, the most common surgical option is high-frequency deep brain stimulation of the subthalamic nucleus or the internal globus pallidus [1–4]. Despite the substantial clinical benefits of surgery, surgical treatment is not without complications, which occur in up to 50% of individuals with PD who undergo deep brain stimulation [2, 5]. These complications include device/surgery-related infections, cognitive decline, depression, speech difficulties, gait disorders, and postural instability [2, 5]. Therefore, there is merit to exploring treatment options that may be used as adjuncts to pharmacologic and surgical treatments prescribed in PD. One such option is exercise, specifically progressive resistance exercise (PRE). 

This review paper will first discuss the rationale for PRE in PD specifically related to bradykinesia and muscle weakness. Then it will review recent findings related to the use of PRE in individuals with PD. Finally, it will identify gaps in knowledge of using PRE in individuals with PD and makes suggestions for future research.

## 2. Rationale for Progressive Resistance Exercise

This section will set up the basis for PRE as a therapeutic intervention in PD. To do so, we will outline the underlying mechanisms for the motor symptoms that can be treated with PRE. We will focus primarily on the central mechanisms that underlie bradykinesia and muscle weakness in PD. Then we will discuss the central changes that accompany PRE and hypothesize how these changes might modify the central mechanisms that underlie bradykinesia and muscle weakness. We will conclude this section with our rationale for the use of PRE in individuals with PD. 

### 2.1. Bradykinesia and Muscle Weakness

Bradykinesia refers to the slowness of a performed movement [6]. Bradykinesia is a primary motor symptom of PD, which is also considered the most functionally debilitating symptom and is a consistent feature of the disease [7]. Muscle weakness, which is a reduction in the amount of force generated by muscle contraction, is often observed in individuals with PD. In fact, several studies have demonstrated that individuals with PD exhibit muscle weakness [8–15]. We have shown that this weakness is exaggerated in the extensor muscles, specifically extensors of the elbow [8, 16]. Additionally, muscle weakness has also been observed across various muscle groups in the trunk [11], upper limbs [14], and lower limbs [9, 10, 13, 14].

In PD, the idea that bradykinesia and weakness are related can be derived from the fact that bradykinesia and muscle weakness might share common underlying mechanisms. Central to the pathophysiology of PD is the known nigral dopaminergic deficit that results in an increase in tonic inhibition of the thalamus and reduction in the excitatory drive to the motor cortex [17]. This, in turn, may result in disruption of the cortical activation of the muscle [18–21] and may manifest as bradykinesia and muscle weakness. Further, muscle power, the product of movement velocity and muscle torque, is reduced in individuals with PD [13]. Also, torque production during isokinetic muscle strength testing in individuals with PD has been shown to vary with movement velocity. Nogaki et al. found that in individuals with PD, no difference was observed in peak torque between the more and the less affected side for slower movements, while for faster movements, the more affected side was significantly weaker than the less affected side [22]. Therefore, reduction in muscle power is indicative of deficits in either strength, movement speed, or both and strengthens the proposed relationship between bradykinesia and muscle weakness. 

Given that the muscle is the final target of cortical output during movement and force production, analyzing the electromyographic (EMG) activation patterns can provide insight into hypothesized impairments that underlie bradykinesia and muscle weakness. We have shown that in individuals with PD, EMG activation patterns during ballistic movements and isometric actions are abnormal and reflect impaired activation of the muscle. Muscle activation patterns during ballistic movement in individuals with PD are abnormal in four significant ways. First, muscle activation patterns show increased variability when compared to age- and sex-matched healthy individuals [23, 24]. Second, in contrast to healthy individuals, the first agonist burst duration does not systematically increase with movement distance [23]. Third, the magnitude of the first agonist burst, early in the disease, is similar to that observed in healthy individuals; however, as the diseases progresses, the magnitude of the first agonist burst is modulated less with increasing movement distance [23]. Fourth, multiple agonist bursting is observed during the acceleration phase of movement, and the number of agonist bursts increases with increasing the movement distance [23, 24]. During isometric actions, individuals with PD manifest deficits throughout the task. At the very beginning of the task, they exhibit decreased rate of torque generation and decreased initial phasic agonist EMG activation, which results in prolonged torque rise times and delayed peak torque [16]. In the middle of the task, during steady-state contraction at 25%, 50%, and 75% of maximal voluntary contraction (MVC), the dominant frequency in the EMG spectrogram in individuals with PD stays fairly constant at ~10 Hz [25]. In healthy individuals, however, the dominant frequency is higher and increases with the increase in isometric torque generation, that is, the dominant frequency shifts from ~18 to 25 Hz when isometric torque generation increases from 25% to 75% of MVC [25]. At the end of the task, the rate of release of muscle contraction is also prolonged, and torque fall times are increased in individuals with PD [26].

The abnormal EMG activation patterns discussed above can be partly explained in terms of an impairment in the corticospinal activation of the muscle, specifically, impairments in variability, intensity, and frequency of the corticospinal activation of the muscle. Increased variability in the corticospinal activation of the muscle could lead to variability in motor unit recruitment and result in increased EMG variability [27]. This increased variability in motor unit recruitment could impair coordinated relaxation of actively contracting motor units, contributing to prolonged deceleration phases during movement and prolonged relaxation times during isometric torque generation. Reduction in the intensity of the corticospinal activation of the muscle [28] may result in impaired motor unit recruitment and could contribute both to bradykinesia and muscle weakness. For instance, impaired motor unit recruitment during movement could result in reduced angular impulse during the acceleration phase of a movement and contribute to bradykinesia, and impaired motor unit recruitment during isometric torque generation could result in reduced peak torque and contribute to muscle weakness. 

Alterations in the frequency of the corticospinal activation of the muscle could also explain some of the abnormal EMG patterns observed in individuals with PD. In healthy subjects the corticospinal activation to the muscle is characterized by three primary frequencies, that is, 10 Hz, 20 Hz, and 40 Hz [29, 30]. The magnetoencephalic (MEG) power spectrum is dominated by ~20 Hz oscillations during weak contractions and ~40 Hz oscillation during strong contractions [29]. Similarly, the mean power in the EMG power spectrum increases from 10 Hz to 25 Hz with increase in percent MVC from 10% to 80% of MVC [31]. In untreated (de novo) individuals with PD relative to age- and sex-matched healthy individuals, resting state cortical activity in the 8–10 Hz band is increased, while activity in the 30–48 Hz band is reduced [32]. Further, in individuals with PD, the EMG power spectrum is dominated by power in the low-frequency band (~10–15 Hz) [25, 26, 29], and the MEG-EMG coherence is strong in this low-frequency band with the MEG signal leading the EMG signal by ~15–38 ms [29]. Thus, one could hypothesize that if the cortical signal to the muscle is dominated by low-frequency oscillations, then this limits the ability to recruit larger, high-frequency motor units, which are required to rapidly generate torque during ballistic movements and generate maximal torque during isometric torque generation. The evidence reviewed in this and the previous two paragraphs suggests that EMG patterns are abnormal in individuals with PD, and one likely explanation for these observed EMG abnormalities is deficits in the variability, intensity, and frequency of the corticospinal activation of the muscle. 

Another factor that could contribute to muscle weakness in individuals with PD is reduced muscle mass. Evidence that muscle mass is reduced in PD is provided by Petroni and colleagues [33]. They reported that midarm muscle circumference was below the 10th percentile in 23% of individuals with advanced PD between 65 and 75 years of age [33]. On the other hand, evidence that this is not the case is provided by Markus and colleagues [34]. They found that even though body mass index and skin fold thickness, relative to age- and sex-matched healthy individuals, were reduced in individuals with PD, midarm circumference was not different from healthy individuals. Thus, the authors concluded that decrease in body mass index was due to a loss of fat and not due to a loss of muscle mass. 

It is important to note that not only does PD cause weakness, but it is highly likely that muscle weakness and functional limitations such as postural instability and gait disturbances lead to reduced physical inactivity as a compensatory mechanism to minimize the likelihood of falls [35]. Therefore, physical inactivity can contribute to muscle weakness and lead to a vicious cycle between muscle weakness and physical inactivity [36].

Even though we cannot discount muscle mass and changes in muscle properties as likely contributors to muscle weakness, it is our stand that the primary contributors to muscle weakness are central in origin and are related to dopaminergic deficits. This is evidenced by the fact that both anti-Parkinsonian medication and deep brain stimulation result in significant improvement in movement speed [24, 37] and significant gains in muscle strength in relatively short amounts of time (not longer than 90 minutes) [16, 38, 39]. Given that the minimum amount of time required to notice appreciable hypertrophy is at least 20 days [40], it is highly unlikely that the immediate strength gains brought about by anti-Parkinsonian medication or deep brain stimulation are caused by gains in muscle mass. 

The question that remains is the extent to which bradykinesia and weakness can be compensated for. We have shown that levodopa and/or deep brain stimulation of the subthalamic nucleus improves bradykinesia and/or muscle strength [24, 38, 39]; however, bradykinesia is not normalized [24, 37]. Moreover, surgical interventions carry significant risks, while medication becomes progressively less effective, and the side effects of medication get progressively worse over time. Therefore, until a cure for PD can be identified, there is a compelling need to develop interventions that improve the signs and symptoms of the disease and slow down the rate at which the signs and symptoms of the disease worsen. One such intervention is PRE, which may be a beneficial and cost effective adjunct treatment in managing PD. As such, if PRE is to be beneficial for individuals with PD, it should bring about central changes that potentially alter nigro-striatal-thalamo-cortical activation and connectivity. Since this has not yet been studied in individuals with PD, we will discuss the central changes that accompany PRE in healthy young and elderly individuals and extend these findings to individuals with PD.

### 2.2. Central Changes That Accompany Progressive Resistance Exercise

The evidence for the central changes that accompany PRE is threefold [41]. First, gains in muscular strength appear before noticeable muscle hypertrophy [41, 42]. After commencing a PRE protocol, strength gains appear as early as 5 days [43], but muscle hypertrophy appears no earlier than 20 days [40]. Therefore, the initial gains in muscle strength cannot be explained by measurable muscle hypertrophy. Instead, a likely explanation for the observed strength gains is the central changes that accompany PRE. Second, cross-education (i.e., improved performance in the untrained limb) is often observed [41]. Munn and colleagues, in their meta-analysis that included 13 studies, concluded that unilateral PRE brings about a 7% increase in strength in the untrained contralateral limb [44]. Given that this cross-education effect is accompanied by increase in muscle surface EMG, but is not accompanied by gains in muscle size, it is likely to be brought about by the central changes that accompany PRE [42, 45]. Third, improvements in performance following PRE are both specific and generalized. The argument for specificity arises from the fact that short-term dynamic strength training results in significantly greater gains in dynamic strength, while isometric strength gains are marginal [46]. While the argument for generalizability arises from the fact that short-term strength training that focuses on increasing isometric strength also improves movement coordination during an untrained task [47]. Thus, both specific and generalizable motor learning effects of PRE provide a third line of evidence for the central changes that accompany PRE.

Further evidence for the central changes that accompany PRE comes from studies employing transcranial magnetic stimulation (TMS), electroencephalography (EEG), functional magnetic resonance imaging (fMRI), and muscle EMG activation patterns. Using TMS, Carroll and colleagues found that for the same level of torque, the amplitude of the motor evoked potential was significantly reduced following a 4-week PRE program [48]. They concluded that resistance training altered the functional properties of the spinal cord circuitry, and fewer motor neurons were recruited for similar levels of pretraining torque. Using EEG, Falvo and colleagues found that the movement-related cortical potentials were significantly attenuated following a 3-week PRE program [49]. They concluded that PRE reduced the neural effort required to move similar levels of pretraining loads. Using fMRI, Liu-Ambrose and colleagues found that in elderly women, following PRE, percent signal change significantly increased in the left anterior insula and the anterior portion of the left middle temporal gyrus [50]. They concluded that PRE could facilitate functional plasticity in the cortex. Using EMG, several studies have shown that muscle activation patterns change after PRE [42, 49, 51–54]. These muscle activation changes following PRE include an increase in the EMG activation [40, 53, 54], possibly due to increased motor unit recruitment [55–57], increased firing rate [57, 58], and improved synchronization [52, 59]; a reduction in the EMG activation to torque ratio, that is, reduction in EMG activation relative to the amount of torque produced [60]; a reduction in the variability associated with the timing, amplitude, and duration of muscle activity [47]; a reduced agonist-antagonist coactivation [61]. In addition, central changes accompanying PRE have been inferred using the H-reflex to examine motor neuron reflex excitability. Holtermann and colleagues found that the amplitude of the H-reflex increased following a 3-week PRE program in healthy individuals [62]. Further, they found that the H-reflex increase in amplitude was associated with an increased rate of force development. This could provide a neurophysiological basis for PRE improving bradykinesia in PD. The exact mechanisms underlying the observed increase of the H-reflex amplitude are not yet known however. The authors suggested that one possibility is that the reflex excitability of the motor neuron pool may be enhanced following PRE.

It should be noted that some of the neural changes discussed in the preceding paragraphs may be affected by factors such as age, sex, the muscle group trained, and their interactions [63, 64]. For instance, following PRE, upper and lower body strength gains are greater in young than in healthy elderly individuals [63]. Also, upper body strength gains are greater in men than in women; however, lower body strength gains are not different between men and women [63].

In summary, PRE can bring about changes throughout the neural axis. Currently, none of the central changes that accompany PRE discussed previously in this section have been researched in individuals with PD. Even though improvements in neuromuscular function have been observed in individuals with PD, from a physiological perspective, further research is required to elucidate the central changes that accompany PRE that could mitigate the motor and nonmotor symptoms observed in PD.

**Figure 1:**
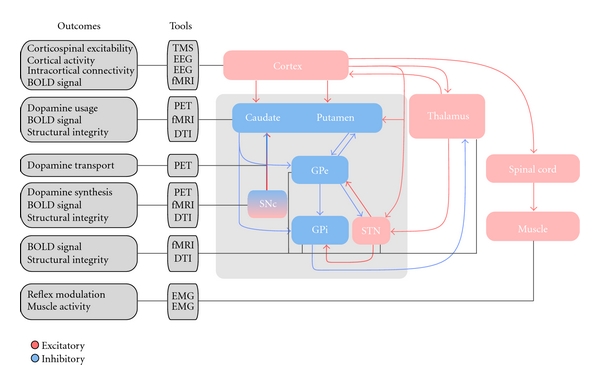
Hypothesized central effects PRE might have in the cortex, basal ganglia, and spinal cord and the tools that can be used to examine these hypothesized changes. TMS, transcranial magnetic stimulation; EEG, electroencephalography; fMRI, functional magnetic resonance imaging; PET: positron emission tomography; DTI: diffusion tensor imaging; EMG: electromyography; SNc: substantia nigra pars compacta; GPe: external globus pallidus; GPi: internal globus pallidus; STN: subthalamic nucleus.

Brain regions where PRE could potentially alter activity include the motor cortex, the posterior putamen, the internal globus pallidus (GPi), and the subthalamic nucleus (STN) (Figure 1). Fisher and colleagues recently demonstrated motor cortical changes following body-weight-supported treadmill training in individuals with PD [65]. They showed that cortical hyperexcitability, which is consistently observed in individuals with PD, is reversed following body-weight-supported treadmill training [65]. Petzinger and colleagues have also shown an increase in the stimulus-evoked dopamine release within the dorsolateral striatum following intensive treadmill training in 1-methyl-4-phenyl-1,2,3,6-tetrahydropyridine- (MPTP-) lesioned mice [66]. Because the dorsolateral striatum is engaged to a high degree during fore- and hind-limb movements during treadmill exercise, they attributed the observed striatal plasticity to use-dependent synaptic plasticity. 

Similarly, there may also be use-dependent synaptic plasticity in the putamen, the GPi, and the STN following PRE. Our lab has conducted a series of studies in which we have shown that nuclei within the basal ganglia scale with the performance of different force producing tasks in both healthy individuals and individuals with PD. Specifically, we have shown that both the globus pallidus and the STN increase percent signal change when generating progressively larger forces in healthy individuals [67]. We have also shown that individuals with PD have a reduced percent signal change in all nuclei of the basal ganglia during an isometric force production task, even early in the disease process when individuals have not yet started their anti-Parkinsonian medication [68]. In addition, blood-oxygen-level-dependent activity in the nuclei of the basal ganglia was correlated to the motor section of the Unified Parkinson's Disease Rating Scale (UPDRS) [69]. The symptom with the highest correlation with basal ganglia activity was bradykinesia. Thus, if PREs were shown to alter the motor section of the UPDRS and bradykinesia, then it is possible that the neuronal activity of the basal ganglia would also be altered by PRE.

Figure 1 illustrates the hypothesized positive effects PRE might have in individuals with PD by possibly altering activity and connectivity in cortical and subcortical regions. It should be noted that these effects of PRE on activity and connectivity in cortical and subcortical regions are purely speculative, as there are no *in vivo* studies that have examined this relationship. As can be clearly seen from the figure however, the basal ganglia are strategically positioned to influence cortical output and modulate control of movement and force. As such, we suggest that one potential reason for why PRE could be therapeutically beneficial for individuals with PD is that it may alter activity in the cortex and the basal ganglia, and connectivity between and within these regions. Advances in experimental techniques, such as TMS, EEG, fMRI, positron emission tomography (PET), diffusion tensor imaging (DTI), and EMG and reflex analyses, afford the possibility of testing hypotheses related to the effect of PRE on neural activity, neural connectivity, and structural integrity *in vivo*, in humans. Figure 1 shows the outcomes and tools that can be used to empirically determine the effects of PRE in specific brain regions. To elaborate, changes in cortical excitability can be measured using TMS, while changes in cortical activity and intracortical connectivity can be measured using EEG. Functional MRI can be used to identify blood-oxygenation-level-dependant signal changes in cortical and subcortical regions following PRE. PET can be used to investigate the effect of PRE on dopamine synthesis, transport, and usage. Diffusion tensor imaging can help elucidate hypotheses related to the changes in structure in cortical and subcortical regions, namely, the substantia nigra, the STN, and the thalamus. Reflex and EMG analyses can be used to identify reflex changes, such as change in H-reflex amplitude, and changes in EMG activation patterns to infer central changes following PRE. Prior to embarking on empirical verification of some of the ideas presented in this paragraph, researchers are cautioned on the technical difficulties, limitations, and the complications of the above-mentioned methods (for a recent detailed review, see Carroll et al. [70]). 

In conclusion, the rationale for the use of PRE in PD is fourfold. First, as discussed above, individuals with PD exhibit muscle weakness. PRE can significantly increase the torque- and power-generating capacity of the muscle, thus directly affecting muscle weakness. Even though other forms of exercise such as aerobic exercise provide substantial health benefits, they do not improve muscle strength by design. Improvements in muscle strength and power have significant impact on bradykinesia [71] and could also facilitate independence in the community, improve functional mobility, and may reduce the risk of falls [72]. Second, exercise interventions in general have been shown to enhance cortical activity, possibly beneficially altering variability, intensity, and frequency components of the corticospinal activation of the muscle [47–49, 73]. This could significantly impact bradykinesia in individuals with PD [65]. Third, exercise may slow down the rate at which the UPDRS scores increase. The UPDRS is the clinical gold standard for assessing the severity and progression of symptoms in PD and for evaluating novel therapies, with higher scores reflecting more severe disease. Reuter and colleagues have shown that a 14-week, intense, multimodal exercise training program can bring about ~12 point reduction in the motor UPDRS scores [74]. Additionally, physical activity has been associated with increasing the survival rate of individuals with PD [75]. Finally, there may well be additional benefits for the non-motor symptoms of PD, such as executive function, mood, and quality of life.

## 3. Progressive Resistance Exercise in PD

Rehabilitation research studies in individuals with PD demonstrate that PRE can have a positive effect on muscle size [76], muscle strength [15, 71, 76–78], muscular endurance [77, 79], and neuromuscular function [71, 76–79]. To date, only one study [76] has quantified changes in muscle size in individuals with PD. Dibble and colleagues observed a 6% increase in muscle volume, measured using volumetric magnetic resonance imaging, after a 12-week eccentric PRE program [76]. Eccentric PRE training involves the use of eccentric muscle activity, that is, the active lengthening of muscles when an external load is imposed; consequently, work is done on the muscle [80]. The rationale used by Dibble and colleagues for using eccentric PRE is that for the same amount of work (i.e., force × distance), high levels of force are generated with minimal oxygen consumption [81]. 

With regard to muscle strength, several studies have demonstrated significant gains in muscle strength following PRE in PD [15, 71, 76–78]. For instance, improvements in strength were observed by Hirsch and colleagues in a randomized controlled trial that compared a 10-week balance training protocol to a 10-week balance training plus PRE protocol [78]. At the end of 10 weeks, they observed significant improvements in strength in knee extension, knee flexion, and ankle plantar flexion in the balance plus PRE group. When the strength measures were combined across the knee and ankle, they observed a 52% increase in strength from before to after treatment in the balance plus PRE group. In another randomized placebo-controlled trial, Hass and colleagues demonstrated significant gains in strength and endurance in upper body muscles, following a 12-week PRE program supplemented with creatine monohydrate [77]. Improvement in endurance was observed by Scandalis and colleagues following an 8-week PRE program that was geared toward the lower body [79]. They found improvements in the total number of abdominal crunches that could be performed at one time. They also observed improvements in lower limb performance, which was quantified as a product of repetitions and weight. Next, we will review the evidence that supports positive changes in neuromuscular function that accompany strength gains in individuals with PD following PRE. 

From a rehabilitation perspective, it is critical that strength gains bring about corresponding improvements in neuromuscular function, such as gait, stair climbing, timed up and go, and postural stability. To this end, recent studies have shown significant improvement in neuromuscular function following PRE interventions in PD. First, improvements in gait have been reported. Three-dimensional gait analyses following an 8-week PRE program demonstrated that individuals with PD increased their gait velocity, stride length, and head angle relative to the floor during midstride [79]. Similar findings of increased gait velocity were also reported by Dibble and colleagues following a 12-week eccentric PRE intervention [71, 76]. The functional gait outcomes included the six-minute walk, ten-meter walk, timed up and go, and stair ascent and descent times. They observed that individuals with PD significantly improved gait velocity and increased the distance walked in six-minutes, reduced the time taken to walk ten meters, reduced the time taken to complete the timed up and go, and reduced stair descent times. Their findings led them to conclude that progressive resistance eccentric exercise could significantly impact bradykinesia. Second, improvement in postural stability has been reported. Hirsch and colleagues showed that individuals with PD demonstrated an improved ability to maintain balance during destabilizing conditions following a 10-week balance plus PRE intervention [78]. Third, improvement in patient-perceived quality of life has been reported. Even though quality of life is not a direct measure of neuromuscular function, it is reasonable to assume that improved neuromuscular function might contribute to improved quality of life. Dibble and colleagues found that eccentric PRE significantly improved patient-perceived quality of life as measured by the Parkinson's disease questionnaire (PDQ-39) [71]. 

In summary, PRE can significantly improve muscle size, muscle strength, muscle endurance, and neuromuscular function and can significantly impact areas often reported to be problematic in individuals with PD, such as bradykinesia, postural instability, and patient-perceived quality of life.

## 4. Limitations of Current Research and Recommendations for Future Research

The few studies that have examined the effect of PRE in PD are no doubt vital to our continued understanding of the effect of PRE and the pursuit of adjunct treatments for PD; however, they are not without limitations. First, it is not clear how anti-Parkinsonian medications interact with PRE. To ascertain the unique contribution of PRE on strength and functional outcomes in PD, it is essential to examine individuals while off anti-Parkinsonian medications. Also, if changes to the underlying disease process are to be evaluated, this is best done while off medication. Among the studies reviewed, all except for Scandalis and colleagues [79] tested individuals with PD while on medication. Thus, more research is required to investigate the unique effect of PRE on outcomes of strength, neuromuscular function, and the underlying disease process. 

Second, the motor UPDRS, which is the clinical gold standard of assessing severity of motor deficits in PD, has rarely been used as an outcome measure while evaluating the effects of PRE. In order to convince neurologists who manage individuals with PD to prescribe exercise as an adjunct therapy, it is vital to demonstrate clinically important change on the motor UPDRS as a result of PRE. Minimal clinically important change on the motor UPDRS is based on the effect of anti-Parkinsonian medication and is defined as a 5-point reduction on the motor UPDRS score [82]. The scores on the motor UPDRS range from 0 to 108, and higher scores indicate more severe motor symptoms. Thus, if exercise can bring about at least a 5-point reduction in the motor UPDRS, one can make a compelling case to include PRE as an adjunct to the standard management of PD. Future research should include the motor UPDRS as an outcome measure while evaluating the effects of PRE. To date, Dibble et al. [71] and Hass et al. [77] have used the motor UPDRS as an outcome measure; however, they both failed to show any clinically relevant change following PRE. This could have been due to the fact that these studies tested individuals with PD while on medication and/or due to the short duration of the PRE intervention. 

Third, long-term effects of PRE are yet to be determined. All of the studies conducted to date evaluate the effect of PRE over 8 to 24 weeks. Given that PD is a progressive neurodegenerative disorder and is further affected by the process of aging, which is accompanied by decline in strength and neuromuscular function [83], it is vital that the long-term effects of PRE are thoroughly understood. For instance, continued benefit of PRE over the long-term could reduce the rate at which the disease progresses. This is significant, especially because recent exciting epidemiological research has concluded that moderate to vigorous levels of physical activity in mid- or later life may be associated with a 40% reduction in the future risk of being diagnosed with PD [84]. Additionally, PRE over the long term could reduce the rate at which dosage of medication is increased and possibly delay the onset of dyskinesias, as well as surgical interventions. Thus, it is essential that future studies evaluate the effects of PRE over the long term in PD. 

Fourth, even though it is accepted that cognitive impairment is frequently observed in PD [85–90], the effect of PRE on cognitive function in PD is not well researched. The rationale for PRE as a therapeutic intervention for cognitive dysfunction is threefold. First, PRE has been found to improve cognitive function in healthy subjects between the age of 65 and 75. Cassilhas et al. demonstrated improved performance on measures of working memory and attention for those assigned to 24 weeks of PRE [91]. More recently, Liu-Ambrose and colleagues demonstrated beneficial cognitive effects of 52 weeks of PRE in community dwelling elderly women [92]. They showed improvements in attention and conflict resolution. Additionally, in a subsequent study with the same sample, they demonstrated changes in percent signal change in brain areas that correspond to conflict resolution [50]. Second, even though aerobic training provides cognitive benefits, a combination of aerobic and PRE has been evidenced to render the greatest cognitive benefits [93]. Recently, two studies have evaluated the combined effect of PRE and aerobic exercise on executive function in PD [94, 95]. Both studies concluded that PRE combined with aerobic exercise improved executive function. Third, there is a strong biological basis for the cognitive benefits gained from PRE. These include the reduction in serum levels of homocysteine [96] and the increase in serum levels of insulin-like growth factor I [97], following PRE, which are both known to be associated with cognitive function [98, 99]. Thus, there is evidence in the literature to support the beneficial effects of PRE on cognitive function, and future research should address this in individuals with PD.

Fifth, the diverse experimental designs employed in the studies reviewed may be less than ideal. Given the realities of conducting research with a patient population, the studies reviewed provide an excellent basis for large-scale, long-term prospective randomized clinical trials. However, the small sample sizes used (between 6 and 14 per group, with a total sample size not exceeding 20), the lack of rater blinding (only Hass et al.'s was a randomized, double-blinded, placebo-controled trial [77]; while Hirsch et al.'s was a randomized control trial, the raters were unblinded [78]), and not employing the intent-to-treat principle in statistical analysis lead to biases that could question the validity of some of the conclusions. Thus, future studies should be blinded, randomized clinical trials, which will provide the most robust experimental design to address the gaps in the literature by assessing the short- and long-term effects of PRE in individuals with PD. 

Sixth, the optimal PRE prescription for individuals with PD is yet to be established. There are two aspects of treatment optimization. The first aspect is the optimization of PRE parameters, such as the frequency, intensity, duration, and mode of exercise (i.e., strength and power training). The second aspect is the optimization of PRE with regards to the various clinical subtypes of PD. Within the general diagnosis of PD, distinct clinical subtypes have been identified based in part on the age of onset, the predominant motor sign (e.g., tremor dominant, nontremor-dominant akinetic rigid etc.), and the clinical course of the disease [100]. There is evidence in the literature that suggests that these different PD subtypes may respond differently to interventions and may progress at different rates [101–103]. For example, individuals who begin with significant rest tremor may not respond as well to levodopa and may progress at a slower rate compared to individuals who present with a nontremor-dominant, akinetic-rigid form of the disease. It is likely that the effect of PRE may vary with the clinical subtype of PD. In addition, the effect of PRE on tremor and rigidity is not yet known. Thus, future research should identify the optimal PRE prescription in the context of the different clinical subtypes of individuals with PD and empirically verify hypotheses related to tremor and rigidity as well.

## 5. Conclusion

In PD, bradykinesia and muscle weakness are primarily due to nigral dopaminergic deficits that alter corticospinal activation. Given the wide array of neural changes that accompany PRE summarized in this paper, the potential to slow the rate of the progression of the symptoms of PD, the improvement in strength and function, and the positive effects on nonmotor symptoms of PD, there is a strong rationale for the use of PRE as an adjunct treatment in PD.
